# Direct comparison of target-reactivity and cross-reactivity induced by CAR- and BiTE-redirected T cells for the development of antibody-based T-cell therapy

**DOI:** 10.1038/s41598-019-49834-2

**Published:** 2019-09-16

**Authors:** Masaki Maruta, Toshiki Ochi, Kazushi Tanimoto, Hiroaki Asai, Takashi Saitou, Hiroshi Fujiwara, Takeshi Imamura, Katsuto Takenaka, Masaki Yasukawa

**Affiliations:** 1 Department of Hematology, Clinical Immunology, and Infectious Diseases, Ehime University Graduate School of Medicine, 791-0295 Toon, Ehime Japan; 2 Division of Immune Regulation, Proteo-Science Center, Ehime University, 791-0295 Toon, Ehime Japan; 3 Department of Molecular Medicine for Pathogenesis, Ehime University Graduate School of Medicine, 791-0295 Toon, Ehime Japan

**Keywords:** Translational immunology, Tumour immunology

## Abstract

The development of chimeric antigen receptor (CAR) and bispecific T-cell engager (BiTE) has led to the successful application of cancer immunotherapy. The potential reactivity mediated by CAR- and BiTE-redirected T cells needs to be assessed to facilitate the application of these treatment options to a broader range of patients. Here, we have generated CAR and BiTE possessing the same single chain fragment variable (scFv) specific for the HLA-A2/NY-ESO-1_157-165_ complex (A2/NY-ESO-1_157_). Using HLA-A2^+^NY-ESO-1^+^ myeloma cells and peptides presented by HLA-A2 molecules as a model, both sets of redirected T cells recognized and killed HLA-A2^+^NY-ESO-1^+^ myeloma cells in an A2/NY-ESO-1_157_-specific manner *in vitro*. Moreover, CAR- and BiTE-activated T cells showed similar functional avidity, as assessed by cytokine production and killing activity, both displaying antitumor reactivity against HLA-A2^+^NY-ESO-1^+^ myeloma cells *in vivo*. Interestingly, cross-reactivity for homologous peptides presented by HLA-A*02:01 and NY-ESO-1_157_ peptide presented by HLA-A2 alleles was not identical between CAR- and BiTE-redirected T cells, probably due to structural differences of modified antibodies. These results have demonstrated that both antitumor CAR- and BiTE-activated T cells have comparable potential to recognize tumors, while paying attention to unknown off-target reactivity that would differ for each antibody-based modality even if the same scFv was employed.

## Introduction

Clinical trials have demonstrated that T-cell therapy can suppress tumor growth even in patients with refractory malignancies^1^. Checkpoint blockade therapy has been shown to induce clinical responses in patients with hematological malignancies such as Hodgkin’s lymphoma, as well as solid tumors^2–6^. However, non-specific activation of patients’ T cells induces not only cytotoxic activity against tumors but also unwanted toxicity against normal tissues, resulting in immune-related adverse events^7–9^. Therefore, immunotherapy utilizing T cells that can accurately segregate tumor cells from normal cells is needed for safe and effective T-cell therapy.

A series of tumor antigens including tumor-specific antigens (TSAs) and tumor-associated antigens (TAAs) have been identified^10^. Peptides derived from these antigens are produced in a proteasome-dependent manner, and are naturally processed and presented by MHC molecules. Antitumor T cells recognize MHC/peptide complexes selectively presented on tumor cells via their T-cell receptor (TCR), and thus exhibit target-specific reactivity. On the basis of this mechanism of T-cell antigen recognition, adoptive transfer therapy has been developed using T cells redirected with TCRs specific for human leukocyte antigen (HLA)/peptide complexes expressed by tumor cells, and clinical trials have shown promise in patients with advanced tumors^11–18^. However, TCR-transduced T cells are laborious to generate, and exhibit cross-reactivity induced by the introduced TCR itself and/or mispairing between the introduced TCR and endogenous TCR^19–21^. To overcome these issues associated with TCR-T cell therapy, recent technical advances have facilitated the generation of antibody-based antitumor receptors that can activate T cells with tumor specificity. For antibody modification, single chain fragment variables (scFvs) generated on the basis of the primary structure of variable regions derived from monoclonal antibodies specific for tumor antigens expressed as surface proteins or HLA/peptide complexes are utilized. Each chimeric antigen receptor (CAR) encodes an scFv followed by a transmembrane domain derived from a co-stimulatory molecule, such as CD28, 4-1BB, and an intracellular domain of the CD3ζ chain^22,23^. One of the bispecific antibodies (BsAb), bispecific T-cell engager (BiTE), possesses an antitumor scFv linked with an anti-human CD3ε scFv^24^. CAR- and BiTE-redirected T cells recognize and kill tumor cells, and clinical trials using CAR- and BiTE-redirected T cells have shown great success in the treatment of refractory CD19^+^ malignancies^25–30^. In contrast, there are not many CAR and BiTE specific for HLA/peptide complexes expressed by refractory tumor cells that can target intracellular antigens^31,32^. To clarify the benefits and risks of antitumor CAR and BiTE, direct comparison of reactivities mediated by CAR- and BiTE-redirected T cells possessing the same scFv recognizing an HLA/peptide complex as a model would reveal in more detail their target-specific reactivity and cross-reactivity by changing specific peptides and HLA alleles. Such knowledge would help facilitate the application of these treatment options to a broader range of patients with refractory malignancies.

Multiple myeloma still remains incurable despite the development of new therapeutic agents^33^. T-cell therapy utilizing CAR and/or BiTE reactive with myeloma cells expressing surface antigens such as BCMA, CD19, CD138, and integrin β_7_ has advanced, and a series of clinical trials have been ongoing^34–37^. Unlike the above surface antigens, NY-ESO-1 is a cancer-testis antigen expressed intracellularly by tumors but not by normal tissues, except for testis lacking the expression of endogenous HLA^38^. Myeloma cells express NY-ESO-1 abundantly, and importantly, an NY-ESO-1_157-165_ peptide (SLLMWITQC) presented by an HLA-A*02:01 molecule (A2/NY-ESO-1_157_) has been demonstrated^39,40^. In a clinical trial, adoptive therapy using T cells modified with TCR specific for A2/NY-ESO-1_157_ in combination with autologous stem cell transplantation successfully induced clinical responses in patients with advanced myeloma^15^. T cells redirected with A2/NY-ESO-1_157_-specific CAR have been established and assessed for their anti-myeloma reactivity^41,42^, although their precise target-specificity and cross-reactivity are still unknown. In addition, A2/NY-ESO-1_157_-specific BiTE has yet to be generated or compared for T-cell reactivity with target cells in comparison with CAR-T cells.

In the present study, to clarify the characteristics of antitumor CAR and BiTE more precisely for expansion of these treatment modalities, we generated both CAR and BiTE carrying the same A2/NY-ESO-1_157_-specific scFv. Employing A2/NY-ESO-1_157_-specific CAR and BiTE and myeloma cells as a model, we investigated the potential target-specific reactivity and cross-reactivity of CAR- and BiTE-redirected T cells for NY-ESO-1_157_ peptide and its homologous peptides presented by the HLA-A2 molecule as well as HLA-A2^+^NY-ESO-1^+^ myeloma cells *in vitro* and *in vivo*, and compared them directly.

## Results

### Target specificity of A2/NY-ESO-1_157_-specific CAR- and BiTE-redirected peripheral blood T cells

**Figure 1:**
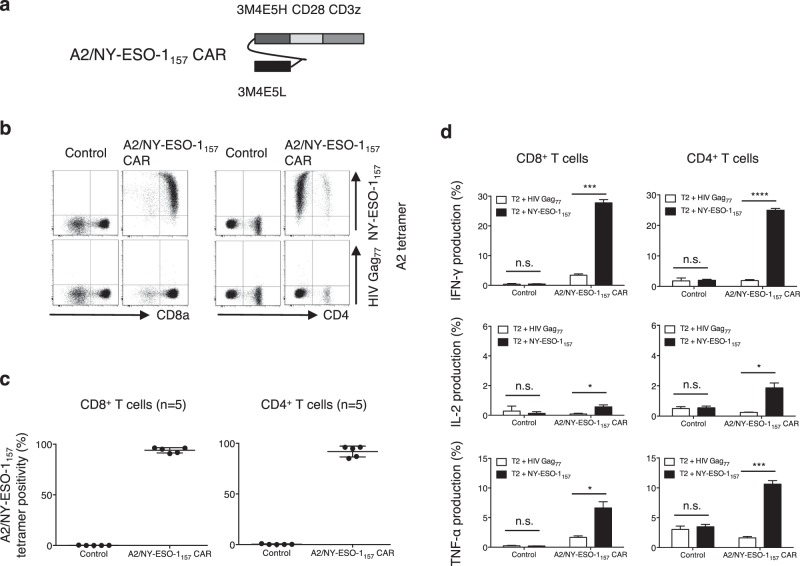
A2/NY-ESO-1_157_-specific CAR redirects peripheral blood T cells. (**a**) Second generation CAR possessing an A2/NY-ESO-1_157_-specific scFv (clone 3M4E5) followed by a transmembrane domain of CD28 and a cytoplasmic domain of CD3ζ was generated. (**b**) The A2/NY-ESO-1_157_-specific CAR was transduced into peripheral blood T cells. These CAR-T cells were stained with 5 μg/mL A2/NY-ESO-1_157_ tetramer or A2/HIV Gag_77_ tetramer. Representative staining of A2/NY-ESO-1_157_ CAR-CD8^+^ T cells (left) and CD4^+^ T cells (right) obtained from donor 1 is shown. (**c**) A2/NY-ESO-1_157_ CAR-CD8^+^ T cells (left) and CD4^+^ T cells (right) derived from 5 out of 5 donors were reproducibly stained with A2/NY-ESO-1_157_ tetramer. (**d**) A2/NY-ESO-1_157_ CAR-CD8^+^ T cells (left) and CD4^+^ T cells (right) established from donor 1 were cocultured with T2 cells loaded with 10 μg/mL NY-ESO-1_157_ peptide or HIV Gag_77_ peptide. The experiments were performed in triplicate and error bars show the SD. *P < 0.05; ***P < 0.001; ****P < 0.0001; n.s., not significant. ΔNGFR-positive cells were gated and analyzed.

First, we generated peripheral blood T cells redirected with A2/NY-ESO-1_157_ CAR. As shown in Fig. 1a, a 2^nd^ generation CAR encoding an A2/NY-ESO-1_157_-specific scFv derived from clone 3M4E5 followed by the CD28 transmembrane domain and cytoplasmic domain of the CD3ζ chain was synthesized. Both A2/NY-ESO-1_157_ CAR-transduced CD8^+^ T cells and CD4^+^ T cells were successfully and reproducibly stained with A2/NY-ESO-1_157_ tetramer (Fig. 1b,c). These CAR-CD8^+^ T cells and CD4^+^ T cells produced multiple cytokines in response to A2/NY-ESO-1_157_ but not A2/HIV Gag_77_ (Fig. 1d). These results indicate that A2/NY-ESO-1_157_ CAR-redirected T cells are able to distinguish NY-ESO-1_157_ peptide from irrelevant peptide and exhibit A2/NY-ESO-1_157_-specific reactivity.

**Figure 2:**
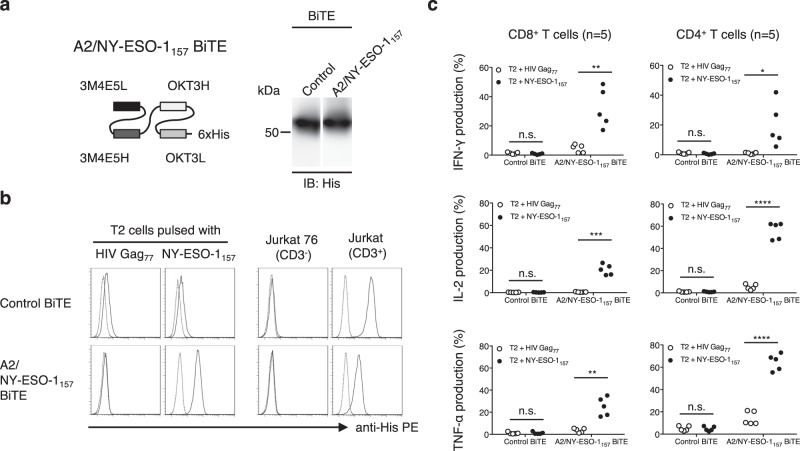
A2/NY-ESO-1_157_-specific BiTE engages peripheral blood T cells with target cells. (**a**) A2/NY-ESO-1_157_-specific BiTE containing an A2/NY-ESO-1_157_-specific scFv (clone 3M4E5) and a human CD3-specific scFv was generated. A hexahistidine (6xHis) tag was inserted at the C-terminus of the construct. Purified 1 μg control BiTE and A2/NY-ESO-1_157_ BiTE were similarly detected by immunoblotting (IB) using anti-His mAb. The full-length blotting image is shown in Supplementary Fig. S4 (top). (**b**) 10 μg/mL A2/NY-ESO-1_157_ peptide or HIV Gag_77_ peptide was loaded onto T2 cells. After floating peptides had been removed, the cells were stained with 10 μg/mL A2/NY-ESO-1_157_ BiTE or control BiTE (left). To see the binding of each BiTE to the CD3 molecule, CD3^+^ Jurkat cells and CD3^−^ Jurkat 76 cells were stained with each BiTE (right). PE-conjugated anti-His mAb was utilized to detect the binding of BiTEs. Dotted lines show the isotype control. (**c**) Freshly isolated peripheral blood T cells derived from 5 different donors were individually incubated with T2 cells pulsed with 10 μg/mL NY-ESO-1_157_ peptide or HIV Gag_77_ peptide in the presence of 1 μg/mL A2/NY-ESO-1_157_ BiTE or control BiTE. Cytokine production by peripheral blood T cells was measured by intracellular cytokine staining via flow cytometry. *P < 0.05; **P < 0.01; ***P < 0.001; ****P < 0.0001; n.s., not significant.

Next, we generated a BiTE, in which an A2/NY-ESO-1_157_-specific scFv was fused with human CD3ε-specific scFv derived from clone OKT3 via a linker sequence (Fig. 2a). The A2/NY-ESO-1_157_-specific BiTE bound to A2/NY-ESO-1_157_, but not to A2/HIV Gag_77_ (Fig. 2b, bottom left). A control BiTE bound to neither A2/NY-ESO-1_157_ nor A2/HIV Gag_77_ (Fig. 2b, top left). On the other hand, both BiTEs bound to CD3^+^ Jurkat cells but not to CD3^−^ Jurkat 76 cells, indicating that the A2/NY-ESO-1_157_ BiTE was able to bind to the CD3 molecule as well as A2/NY-ESO-1_157_ on the cell surface (Fig. 2b, right). We also tested whether freshly isolated peripheral blood T cells recognized A2/NY-ESO-1_157_ in the presence of the A2/NY-ESO-1_157_ BiTE. Peripheral blood CD8^+^ T cells and CD4^+^ T cells derived from 5 out of 5 donors we tested appeared to significantly produce a panel of cytokines against A2/NY-ESO-1_157_ in the presence of the A2/NY-ESO-1_157_ BiTE, but not the control BiTE (Fig. 2c). These results clearly indicate that the A2/NY-ESO-1_157_ BiTE successfully redirected peripheral blood T cells with A2/NY-ESO-1_157_ specificity. Importantly, a BiTE appeared to be unable to activate T cells only by binding to the CD3 molecule, suggesting that engagement of T cells with target cells via BiTE is necessary for stimulation of T cells.

### A2/NY-ESO-1_157_ CAR- and BiTE-redirected T cells recognize HLA-A2^+^NY-ESO-1^+^ myeloma cells

**Figure 3:**
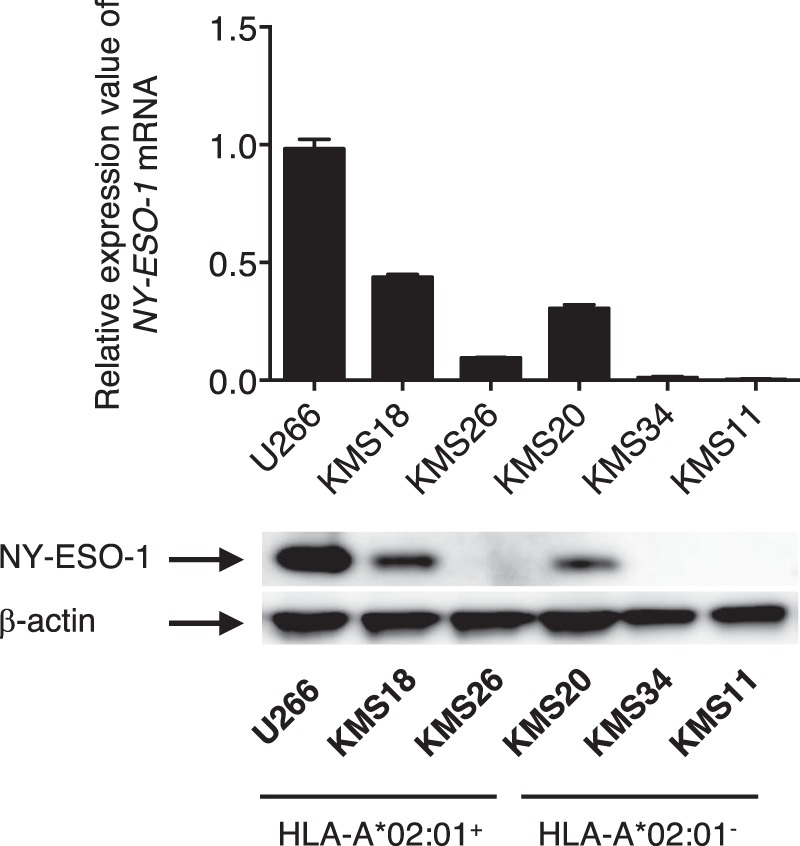
Myeloma cells express NY-ESO-1. Expression of *NY-ESO-1* mRNA and NY-ESO-1 protein was measured by qRT-PCR (top) and Western blotting (bottom). Data were normalized using *hHPRT1* for qRT-PCR and β-actin for Western blotting. The expression of *NY-ESO-1* mRNA in U266 cells is shown as 1.0, and the expression levels in other cells are calculated relative to this value. Error bars show the SD. Among six myeloma cell lines we tested, three were HLA-A*02:01-positive, and three were HLA-A*02:01-negative, as indicated at the bottom. The full-length blotting images are displayed in Supplementary Fig. S4 (bottom).

**Figure 4:**
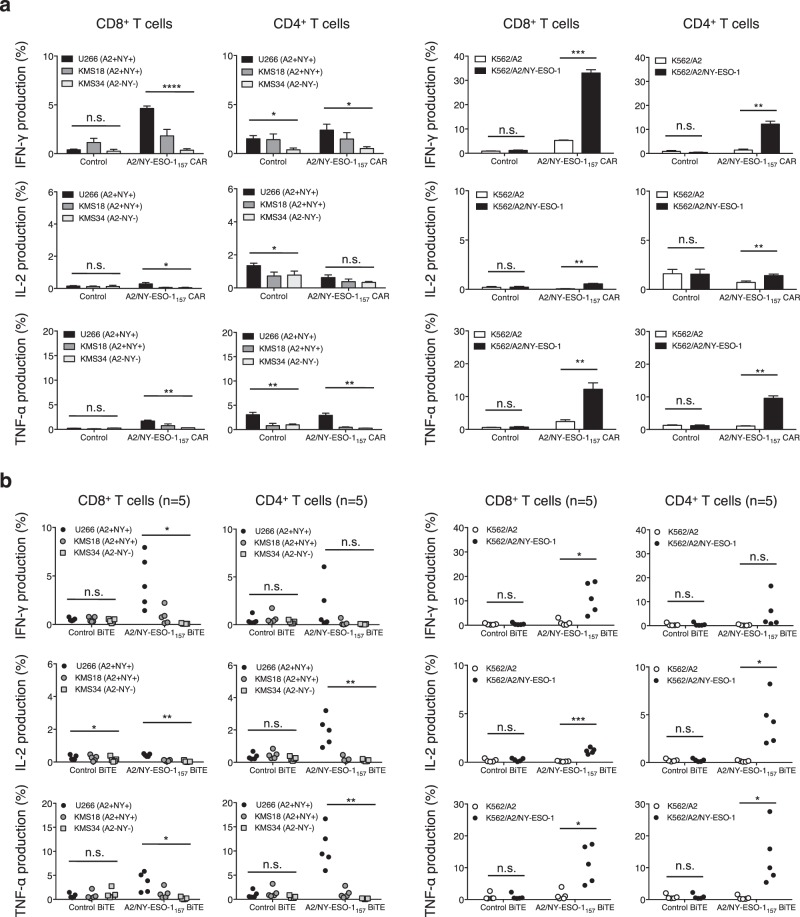
A2/NY-ESO-1_157_ CAR- and BiTE-redirected T cells recognize myeloma cells in an A2/NY-ESO-1_157_-specific manner. (**a**) A2/NY-ESO-1_157_ CAR-transduced CD8^+^ T cells and CD4^+^ T cells were incubated with the indicated target cells, and their cytokine production was measured by intracellular cytokine assay. The HLA-A2 (A2) and NY-ESO-1 (NY) positivity of each myeloma cell line used is also shown. The experiments were performed in triplicate, and ΔNGFR-positive cells were gated and analyzed. The experiments were repeated twice, and representative data obtained from donor 1 are shown. Error bars depict the SD. (**b**) Freshly isolated peripheral blood T cells derived from 5 different donors were incubated with the indicated target cells in the presence of 5 μg/mL A2/NY-ESO-1_157_ BiTE or control BiTE. Cytokine production was assessed by intracellular cytokine staining. *P < 0.05; **P < 0.01; ***P < 0.001; ****P < 0.0001; n.s., not significant.

Next, we investigated whether A2/NY-ESO-1_157_ CAR- and BiTE-redirected T cells recognize target cells endogenously expressing A2/NY-ESO-1_157_. NY-ESO-1 expression of six myeloma cell lines expressing or not expressing HLA-A*02:01 was examined. Three out of six myeloma cell lines we tested overexpressed *NY-ESO-1* mRNA and NY-ESO-1 protein (Fig. 3). A2/NY-ESO-1_157_ CAR-CD8^+^ T cells and CD4^+^ T cells recognized HLA-A2^+^NY-ESO-1^+^ U266 cells, but neither HLA-A2^+^NY-ESO-1^−^ KMS26 cells nor HLA-A2^−^NY-ESO-1^−^ KMS34 cells (Supplementary Fig. S1). It appeared that the target-specific cytokine release occurred mainly from CAR-transduced CD8^+^ T cells, suggesting that binding of the CD8 molecule to the HLA class I molecule is able to enhance the cytoplasmic signals of the CAR-T cells (Fig. 4a, left). Peripheral blood CD8^+^ T cells and CD4^+^ T cells significantly produced cytokines against U266 cells in the presence of A2/NY-ESO-1_157_ BiTE (Fig. 4b, left and Supplementary Fig. S1).

**Figure 5:**
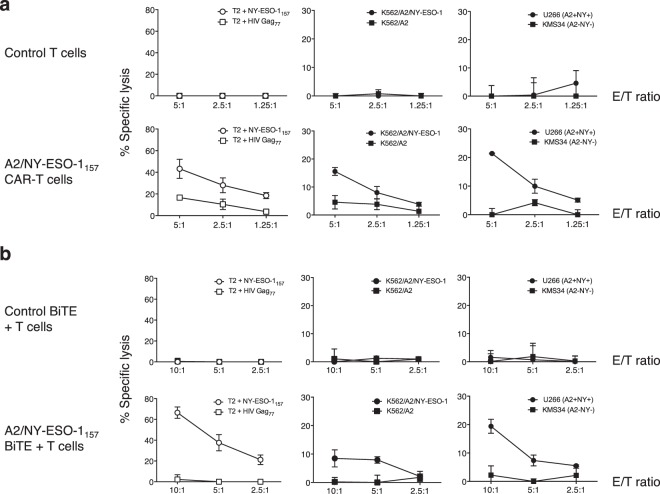
A2/NY-ESO-1_157_ CAR- and BiTE-redirected T cells kill HLA-A2^+^NY-ESO-1^+^ target cells *in vitro*. (**a**) A2/NY-ESO-1_157_ CAR-transduced T cells were incubated with ^51^Cr-labeled T2 cells pulsed with 10 μg/mL NY-ESO-1_157_ peptide or HIV Gag_77_ peptide (left). These responder cells were also cocultured with ^51^Cr-labeled K562 transfectants (middle) or myeloma cell lines (right) at the indicated E/T ratio. (**b**) Freshly isolated peripheral blood T cells were also incubated with ^51^Cr-labeled target cells as described above in the presence of A2/NY-ESO-1_157_ BiTE or control BiTE (1 μg/mL for peptide-pulsed target cells or 5 μg/mL for other target cells) at the indicated E/T ratio. All the experiments were performed in triplicate and error bars depict the SD. The results obtained from donor 1 are displayed.

We also assessed whether CAR- and BiTE-redirected T cells indeed recognize naturally processed and presented A2/NY-ESO-1_157_ in target cells. For this purpose, K562 cells, which lack expression of endogenous HLA and NY-ESO-1, were transduced with the HLA-A*02:01 gene with or without the *NY-ESO-1* gene. The level of HLA-A2 expression was similar among K562/A2, K562/A2/NY-ESO-1, and U266 cells; on the other hand, NY-ESO-1 expression by K562/A2/NY-ESO-1 cells was higher than that by U266 cells (Supplementary Fig. S2). Cytokine production by CAR- and BiTE-redirected CD8^+^ T cells and CD4^+^ T cells against K562/A2/NY-ESO-1 cells was more abundant in comparison to that against U266 cells (Fig. 4). Importantly, CAR- and BiTE-redirected CD8^+^ T cells and CD4^+^ T cells segregated K562/A2/NY-ESO-1 cells from K562/A2 cells (Fig. 4a,b, right and Supplementary Fig. S1). We also confirmed that CAR- and BiTE-redirected T cells killed NY-ESO-1_157_ peptide-pulsed T2 cells, K562/A2/NY-ESO-1 cells, and HLA-A2^+^NY-ESO-1^+^ U266 cells, but not other control cells (Fig. 5). Cytotoxicity against HLA-A2^+^NY-ESO-1^+^ myeloma cells mediated by CAR-T cells was more efficient than that mediated by BiTE-redirected T cells *in vitro*, although it must be noted that we employed freshly isolated peripheral blood T cells for experiments using BiTEs. These results indicate that BiTE-redirected T cells as well as CAR-redirected T cells possess sufficient avidity to recognize endogenously processed and presented A2/NY-ESO-1_157_, resulting in cytokine production and specific killing of HLA-A2^+^NY-ESO-1^+^ myeloma cells.

### A2/NY-ESO-1_157_ BiTE-redirected T cells as well as CAR-T cells show sufficient functional avidity to kill HLA-A2^+^NY-ESO-1^+^ myeloma cells *in vivo*

**Figure 6:**
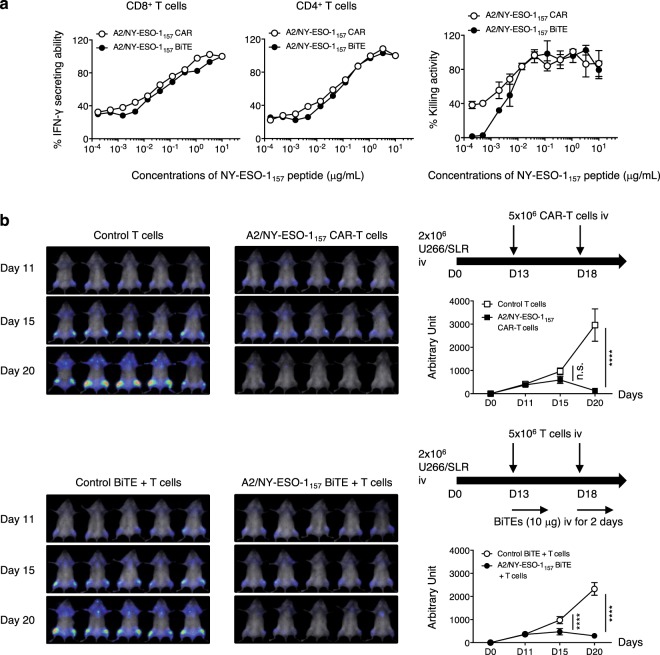
A2/NY-ESO-1_157_ CAR- and BiTE-redirected T cells show sufficient functional avidity to induce anti-myeloma reactivity *in vivo*. (**a**) Functional avidities of A2/NY-ESO-1_157_ CAR- and BiTE-redirected CD8^+^ T cells and CD4^+^ T cells were assessed using T2 cells pulsed with graded concentrations of NY-ESO-1_157_ peptide as stimulator cells. Side-by-side experiments using similarly activated T cells derived from donor 1 as responder cells were performed. CAR- and BiTE-redirected T cells were incubated with target cells for 16 hours after adding BFA to measure IFN-γ production, and for 5 hours to examine target-specific lysis, respectively. % IFN-γ secreting ability (left, middle) and % killing activity (right) of CAR- and BiTE-redirected T cells are shown. The reactivity of responder cells against T2 cells pulsed with 10 μg/mL (IFN-γ) or 1 μg/mL (killing) NY-ESO-1_157_ peptide is considered as 100%, and each reactivity is calculated relative to this value. The experiments were repeated twice, and representative data are shown. Error bars show the SD. (**b**) Two million U266/SLR cells were intravenously injected into NOG mice. After engraftment of U266/SLR cells had been confirmed by bioluminescence imaging assays on Day 11, 5.0 × 10^6^ CAR-T cells or control T cells were injected twice on Days 13 and 18 (top). Similarly activated bulk T cells were injected twice on Days 13 and 18 followed by intravenous injection of 10 μg A2/NY-ESO-1_157_ BiTE or control BiTE on Days 13, 14, 18, and 19 (bottom). These experiments were performed side-by-side. Measured photon counts obtained from a mouse in both the prone and supine positions were aggregated, and calculated as arbitrary units for comparison between the groups. The experiments were repeated twice using distinct donor-derived T cells (donor 1), and representative data are displayed. Error bars indicate the SD. ****P < 0.0001; n.s., not significant.

Since we detected sufficient cytokine production and specific lysis mediated by CAR- and BiTE-redirected T cells against endogenously expressed A2/NY-ESO-1_157_ in target cells, we directly compared the functional avidity of CAR-redirected T cells with that of BiTE-redirected T cells. To compare this under the same conditions, we prepared similarly activated T cells derived from the same donor as responder cells for the CAR and BiTE experiments. The functional avidity of BiTE-redirected CD8^+^ T cells and CD4^+^ T cells measured in terms of cytokine production and killing activity was approximately similar to that of CAR-redirected CD8^+^ T cells and CD4^+^ T cells (Fig. 6a). We then compared the *in vivo* antitumor effects of CAR-redirected T cells with that of BiTE-redirected T cells. CAR- and BiTE-redirected T cells with a similar CD4/CD8 ratio were prepared for *in vivo* side-by-side experiments (Supplementary Fig. S3). Using *in vivo* bioluminescence imaging assays, we confirmed that U266 cells were successfully engrafted in NOG mice on Day 11. On Day 13 and Day 18, CAR-T cells or control T cells were injected intravenously into tumor-bearing mice. The same number of similarly activated T cells were administered to NOG mice followed by intravenous injection of an A2/NY-ESO-1_157_ BiTE or a control BiTE for direct comparison. On Day 20, tumor suppression was achieved by treatment with A2/NY-ESO-1_157_ CAR-T cells but not control T cells. Antitumor effects induced by responder cells were obtained with the A2/NY-ESO-1_157_ BiTE, but not the control BiTE (Fig. 6b). On Day 15, tumor growth was significantly suppressed by treatment with the T cells in combination with A2/NY-ESO-1_157_ BiTE, but not with CAR-T cells. In contrast, on Day 20, there was a tendency for CAR-T cells to suppress tumor growth more effectively (Fig. 6b). Because of xenoreactivity, we were unable to observe antitumor effects of both sets of redirected T cells for over 20 days in our experiments. However, these results suggest that CAR- and BiTE-redirected T cells successfully kill HLA-A2^+^NY-ESO-1^+^ tumors *in vivo*. BiTE is able to engage T cells with tumor cells and induce antitumor responses at tumor sites in the early stage.

### A2/NY-ESO-1_157_ CAR- and BiTE-redirected T cells possess potent cross-reactivity

**Figure 7:**
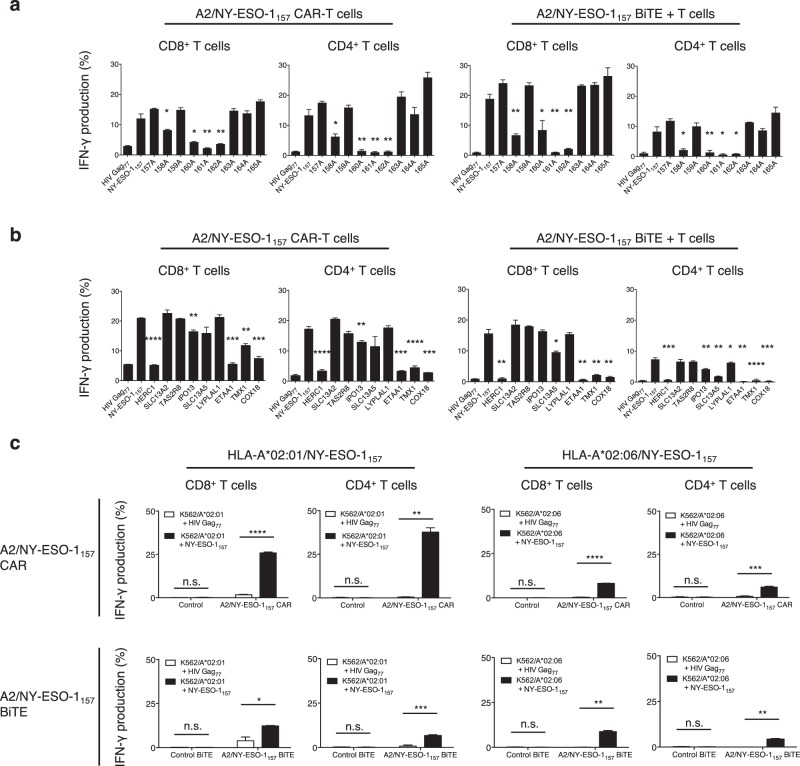
A2/NY-ESO-1_157_ CAR- and BiTE-redirected T cells show potential cross-reactivity. (**a**) A2/NY-ESO-1_157_ CAR-transduced CD8^+^ T cells and CD4^+^ T cells were incubated with T2 cells pulsed with 9 different alanine-substituted peptides at 5 μg/mL (left). Similarly, freshly isolated peripheral blood T cells were cocultured with T2 cells loaded with 5 μg/mL alanine-substituted peptides in the presence of 1 μg/mL A2/NY-ESO-1_157_ BiTE (right). IFN-γ production (%) was measured using intracellular cytokine assays. Each response for alanine-substituted peptides was compared with that for the original NY-ESO-1_157_ peptide. *P < 0.05; **P < 0.01. (**b**) A2/NY-ESO-1_157_ CAR-T cells (left) or BiTE-redirected T cells (right) were stimulated with T2 cells individually pulsed with 10 μg/mL irrelevant HIV Gag_77_ peptide, original NY-ESO-1_157_ peptide, and 9 different peptides homologous to NY-ESO-1_157_. IFN-γ production (%) was examined similarly to (**a**). *P < 0.05; **P < 0.01; ***P < 0.001; ****P < 0.0001. (**c**) A2/NY-ESO-1_157_ CAR-transduced CD8^+^ T cells and CD4^+^ T cells were stimulated with K562/A*02:01 or K562/A*02:06 cells pulsed with 10 μg/mL NY-ESO-1_157_ peptide or HIV Gag_77_ peptide (top). Freshly isolated peripheral blood T cells were also incubated with the same targets in the presence of 10 μg/mL NY-ESO-1_157_ BiTE or control BiTE (bottom). IFN-γ production (%) by T cells was similarly examined. *P < 0.05; **P < 0.01; ***P < 0.001; ****P < 0.0001; n.s., not significant. All the experiments from (**a**) to (**c**) were performed in triplicate, and ΔNGFR-positive cells among CAR-T cells were gated and analyzed. The experiments were repeated twice, and representative data obtained from donor 1 are displayed. Error bars depict the SD.

Clinical trials have indicated the risks of cross-reactivity of gene-modified antitumor T cells with normal cells^19,20^. The cross-reactivity of CAR- and BiTE-redirected T cells has also been suggested^21^; however, their characteristics have yet to be investigated in detail. Therefore, we examined the potential cross-reactivity of A2/NY-ESO-1_157_ CAR- and BiTE-redirected T cells using peptides homologous to NY-ESO-1_157_. CAR- and BiTE-redirected T cells derived from the same donor were prepared. A single alanine substitution in the NY-ESO-1_157_ peptide was performed to determine the important amino acids recognized by CAR- and BiTE-redirected T cells. Since the responsiveness of these T cells to peptides in which the second, fourth, fifth, and sixth amino acids were substituted was reduced, as shown in Fig. 7a, we searched for homologous peptides containing the xLxMWIxxx sequence using the ScanProsite tool. Nine different peptides homologous to the original NY-ESO-1_157_ were synthesized (Supplementary Table S1). A2/NY-ESO-1_157_ CAR- and BiTE-redirected T cells responded differently to some of them. In addition, CAR- and BiTE-redirected CD8^+^ T cells showed a tendency to be more cross-reactive than CD4^+^ T cells. Although proteins containing cross-reactive peptides recognized by A2/NY-ESO-1_157_ scFv, such as SLC13A2, TAS2R8, LYPLAL1 as shown in Fig. 7b, are likely to be expressed in normal tissues, their expression level does not appear to be higher than that of NY-ESO-1 expressed by tumor cells, at least on the basis of an *in silico* search. Moreover, it is unknown whether these peptides with HLA-A*02:01 could be naturally processed and presented by normal tissues.

We also tested the cross-reactivity of CAR- and BiTE-redirected T cells with NY-ESO-1_157_ peptide presented by different HLA-A2 alleles. In this study, an HLA-A*02:06 molecule was employed, as NY-ESO-1_157_ peptide can bind to HLA-A*02:06 as well as to HLA-A*02:01^18^. A2/NY-ESO-1_157_ CAR-T cells recognized NY-ESO-1_157_ peptide pulsed onto HLA-A*02:01 and HLA-A*02:06 with different magnitude (Fig. 7c, top). Interestingly, peripheral blood T cells were reactive with NY-ESO-1_157_ peptide presented by HLA-A*02:01 and HLA-A*02:06 in the presence of A2/NY-ESO-1_157_ BiTE comparably (Fig. 7c, bottom). Although collectively, cross-reactivity for HLA-A2/peptide complexes would aid the application of these treatment modalities to both HLA-A*02:01- and HLA-A*02:06-positive patients, careful attention to any unknown off-target reactivity with normal tissues will be necessary.

## Discussion

Since previous studies had employed scFvs recognizing surface antigens that were not HLA/peptide complexes, comparison of target-specific reactivity and cross-reactivity mediated by CAR- and BiTE-redirected T cells was difficult. To our knowledge, this is the first study to have precisely assessed target-specific reactivity and cross-reactivity of CAR-T cells and BiTE-redirected T cells side-by-side, especially using the same A2/NY-ESO-1_157_-specific scFv and specific peptides presented by HLA-A2 molecules as a model. CAR- and BiTE-redirected T cells recognized naturally processed and presented A2/NY-ESO-1_157_ and killed HLA-A2^+^NY-ESO-1^+^ myeloma cells in an A2/NY-ESO-1_157_-specific manner. In our experiments, CAR- and BiTE-redirected T cells showed similar functional avidity, as assessed by cytokine production and killing activity, and thereby both displayed cytotoxicity for HLA-A2^+^NY-ESO-1^+^ myeloma cells *in vivo*. Cross-reactivity for homologous peptides presented by HLA-A*02:01 and NY-ESO-1_157_ peptide presented by HLA-A2 alleles was not identical between CAR- and BiTE-redirected T cells.

Stone and colleagues have prepared CAR and BiTE possessing the same scFv specific for a glycopeptide epitope presented by murine fibrosarcoma cells^43^. They quantified the number of epitopes on the surface of target cell lines using a monoclonal antibody, and compared the cytokine production capacity and killing activity of CAR- and BiTE-redirected T cells against a couple of target cell lines presenting different amounts of glycopeptide epitopes. They concluded that CAR-transduced T cells have greater sensitivity than BiTE-treated T cells *in vitro*. Hoseini and colleagues have also investigated the functional capabilities of T cells redirected with CAR or BsAb possessing the same scFv specific for disialoganglioside GD2. Using similarly activated T cells, they found that BsAb-redirected T cells show greater survival and rapidly induce tumor regression *in vivo* in comparison with CAR-T cells^44^. When we precisely measured the functional avidity of CAR- and BiTE-redirected T cells using similarly activated T cells as responder cells and graded the concentrations of NY-ESO-1_157_ peptide, both sets of redirected T cells showed approximately similar avidity, resulting in sufficient reactivity against HLA-A2^+^NY-ESO-1^+^ myeloma cells *in vivo*. Interestingly, antitumor effects induced by BiTE-redirected T cells were seen in the early phase relative to those induced by CAR-T cells. A2/NY-ESO-1_157_ tetramer staining of CAR-T cells ranged from bright to dim (Fig. 1b), whereas BiTE was able to engage infused T cells with tumor cells equivalently. In addition, engagement of T cells with tumor cells by BiTE induces cytolytic synapses similar to those formed by binding of TCRs to HLA/peptide complexes, relative to those induced by CAR-T cells^45,46^. These characteristics may account for the difference in antitumor effects between BiTE-redirected T cells and CAR-transduced T cells in the early stage. On the other hand, CAR-T cells had a tendency to show delayed antitumor responses when compared with BiTE-activated T cells, suggesting that in the context of optimal delivery of intravenously injected BiTE into tumor sites to engage T cells with tumor cells *in vivo*, BiTE-redirected T cells have the potential to suppress myeloma, which is a slowly progressive disease. These factors should be considered when applying BiTE to patients, even though CAR- and BiTE-activated T cells display similar functional avidity.

CAR- and BiTE-redirected T cells differed in their recognition of NY-ESO-1_157_ peptide bound to an HLA-A*02:01 or HLA-A*02:06 molecule. This type of cross-reactivity allows us to broaden the range of patients who can be treated with T cells redirected with CAR or BiTE specific for HLA/peptide complexes. On the other hand, these cells showed different degrees of cross-reactivity with some homologous peptides presented by HLA-A*02:01. Previous studies have revealed that scFvs showing cross-reactivity with different peptides presented by an HLA molecule show atypical orientations, tilting toward α1-helix or α2-helix and forming few direct contacts with the peptide^47^. However, this A2/NY-ESO-1_157_-specific scFv was obviously dependent on homologous peptides as well as NY-ESO-1_157_ peptide along with an HLA-A2 allele^48^, suggesting that an A2/NY-ESO-1_157_ scFv cross-reactive with HLA-A2/peptide complexes adopts conformations differing from scFvs possessing high cross-reactivity^47^. The difference in the magnitude of cross-reactivity of CAR- and BiTE-redirected T cells might be explained by the structures of CAR and BiTE, which are affected by the transmembrane/intracellular domain composed of a CAR or a CD3-specific scFv containing a BiTE. This means that other types of modified antibodies containing the same scFv might display different cross-reactivity with target antigens. Crystal structure analyses of CAR and BiTE possessing an identical scFv for the target antigens would be warranted to clarify the underlying mechanism.

CAR- and BiTE-redirected CD8^+^ T cells showed a greater tendency to possess enhanced target-specific reactivity and cross-reactivity than CD4^+^ T cells. Some studies have demonstrated that T-cell cross-reactivity as well as target-specific reactivity is enhanced by binding of CD8 to the HLA class I molecule^49,50^. Although we employed an scFv targeting an HLA class I/peptide complex, CD8^+^ T cells redirected with CAR or BiTE possessing scFvs specific for other surface antigens may also have displayed enhanced reactivity due to interaction between CD8 and HLA class I without target-specific binding of an scFv to HLA/peptide complexes. Further studies of this issue will be needed.

Our study has revealed the target-specific reactivity of CAR and BiTE and the need for caution regarding unknown off-target adverse events caused by cross-reactivity of CAR, BiTE, or other antibody-based modalities. The clinical application of antibody-based T-cell therapy can be expanded, since scFvs can be broadly applied to both cell therapy and other biological reagents, unlike other T-cell therapies. Moreover, using flow cytometry or immunohistochemistry, scFvs that bind to an HLA/peptide complex can be utilized to assess whether target HLA/peptide complexes are naturally processed and presented by tumor cells before the introduction of T-cell therapy. This would make it possible to select patients who would benefit most from those therapies. For clinical application and diagnostics, optimization of A2/NY-ESO-1_157_ scFv will be necessary. However, the most important outcome of this study is that, using an A2/NY-ESO-1_157_ scFv and myeloma cells as a model, we have demonstrated both the capacity and the risk of antitumor CAR- and BiTE-redirected T cells by direct comparison for further development of antibody-based T-cell therapy.

## Materials and Methods

### Cells

K562 cells, which lack expression of endogenous HLA and NY-ESO-1, were purchased from the American Type Culture Collection (ATCC) and cultured with RPMI1640 supplemented with 10% fetal calf serum (FCS). T2 cells endogenously expressing HLA-A*02:01 as one of the HLA alleles were cultured with RPMI1640 supplemented with 10% FCS. The U266 cell line was a generous gift from Dr. Ryosuke Uchibori and Dr. Keiya Ozawa, Jichi Medical University. Other myeloma cell lines were kindly provided by Dr. Takemi Ohtsuki, Kawasaki Medical University. Myeloma cell lines were cultured in RPMI1640 supplemented with 10% FCS. Plat-A cells (kindly provided by Dr. Toshio Kitamura, Institute of Medical Sciences, University of Tokyo) were maintained in DMEM supplemented with 10% FCS, 1 μg/mL puromycin and 10 μg/mL blasticidin, as described previously^51^. PG13 cells were cultured in DMEM supplemented with 10% FCS. Jurkat 76 cells, which lack the expression of endogenous TCR and CD3 (generous gift from Dr. Mirjam Heemskerk, Leiden University Medical Center) were cultured in RPMI1640 supplemented with 10% FCS^52^. Human peripheral blood mononuclear cells (PBMCs) were isolated from healthy volunteers using Ficoll-Paque (GE Healthcare) and stored until use. All the experiments using human samples were approved by the Certified Review Board, Ehime University. All methods were performed in accordance with the relevant guidelines and regulations. Written informed consent was given by all donors.

### Genes

Variable regions derived from immunoglobulin light and heavy chains of an HLA-A*02:01-restricted NY-ESO-1_157-165_-specific monoclonal antibody (mAb) (clone: 3M4E5) were linked with a linker sequence to generate the scFv^48,53^. A second generation *A2/NY-ESO-1*_157_ *CAR* gene encoding the scFv followed by a transmembrane region of CD28 and an intracellular domain of the CD3ζ chain was synthesized by GeneArt (Thermo Fisher Scientific). To detect CAR-transduced T cells, a *CAR* gene was fused with a truncated *nerve growth factor receptor* (*ΔNGFR*) gene via a furin cleavage site, an SGSG spacer sequence, and a codon-optimized P2A sequence^54–56^. A *BiTE* gene was also generated by GeneArt. A human CD3ε-specific scFv derived from a CD3ε-specific mAb (clone: OKT3) was fused with an A2/NY-ESO-1_157_-specific scFv or an anti-human IgG scFv for control via an SGSG linker sequence. A hexahistidine (6xHis) tag was inserted at the C-terminus of the construct with an SGSG linker sequence for BiTE purification and detection. All genes were individually integrated into a pMX retroviral vector for transduction^57^.

### Transfectants

The experiments were reviewed and approved by the institutional review board at Ehime University. K562 cells were transduced with the HLA-A*02:01 or HLA-A*02:06 gene to establish K562/A*02:01 (K562/A2) or K562/A*02:06 cells. In some experiments, K562/A2 cells were transduced with the *NY-ESO-1* gene to establish K562/A2/NY-ESO-1 cells. U266 cells were transduced with the luciferase gene (SLR: stable luciferase red) to establish U266/SLR cells for *in vivo* bioluminescence imaging assays. Human peripheral blood T cells were stimulated with 100 IU/mL human IL-2 and 50 ng/mL anti-human CD3ε mAb (clone OKT3) for 2 days before transduction. Then, T cells were cultured in RPMI1640 supplemented with 10% AB type human serum (Sigma Aldrich) and retrovirally transduced with an *A2/NY-ESO-1*_157_ *CAR* gene or a control gene (*ΔNGFR* alone) to establish gene-modified T cells as described previously^55,56^. To perform the ELISA experiments, NGFR-positive T cells labeled with FITC-conjugated anti-human NGFR mAb (clone ME20.4) were collected by FACSAria II (Becton Dickinson). When needed, further selection of CD8- or CD4-positive T cells was performed using microbeads (Miltenyi Biotec). To confirm the killing activity of CAR-T cells *in vitro* and *in vivo*, gene-modified T cells were isolated using PE-conjugated anti-human NGFR mAb (clone ME20.4) in combination with anti-PE microbeads (Miltenyi Biotec). 293 T cells were retrovirally transduced with an *A2/NY-ESO-1*_157_ *BiTE* gene or a control *BiTE* gene to produce supernatants containing BiTE. The BiTEs were purified using a His GraviTrap column and a Vivaspin column (GE Healthcare) for the following experiments.

### Flow cytometry

T cells were stained with PC5-conjugated anti-human CD8 mAb (clone B9.11), FITC-conjugated anti-human CD4 mAb (clone OKT4), and V450-conjugated anti-human NGFR mAb (clone C40-1457). Biotinylated HLA-A*02:01/NY-ESO-1_157_ monomer and HLA-A*02:01/HIV Gag_77_ monomer (MBL) were multimerized using PE-conjugated streptavidin (Thermo Fisher Scientific), and utilized for detection of CAR-transduced T cells as described elsewhere^55,56^. Myeloma cell lines and K562 transfectants were stained with PE-conjugated anti-HLA-A2 mAb (clone BB7.2). They were also stained with anti-human NY-ESO-1 mAb (clone E978) and PE-conjugated AffiniPure F(ab’)_2_ goat anti-mouse IgG (Fcγ specific) (Jackson ImmunoResearch). To confirm target-specific binding of BiTE, target cells were stained with purified BiTE in combination with PE-conjugated anti-His mAb (clone GG11-8F3.5.1) for detection. Human CD3^+^ T cells were collected from freshly isolated PBMCs with anti-CD3 microbeads (Miltenyi Biotec) for *in vitro* experiments using BiTEs unless otherwise specified. To investigate A2/NY-ESO-1_157_-specific cytokine production by CAR- and BiTE-redirected T cells, 3.0 × 10^5^ responder cells were incubated with 5.0 × 10^4^ target cells. Brefeldin A (BFA) was added after 2 hours, and the cells were incubated for an additional 4 hours for CAR-T cells and 16 hours for BiTE-redirected T cells to produce cytokines, unless otherwise specified. Then, PE-conjugated anti-human TNF-α mAb (clone Mab11), APC-conjugated anti-human IL-2 mAb (clone MQ1-17H12), and PC7-conjugated anti-human IFN-γ mAb (clone B27) together with other surface markers as described above were utilized for detection. All samples were analyzed using a Gallios flow cytometer (Beckman Coulter) and FlowJo Version 7.6.5 software (Becton Dickinson). ΔNGFR-positive cells were gated to assess CAR-T cells.

### Enzyme-linked immunosorbent assays (ELISA)

IFN-γ production by 1.0 × 10^5^ CAR- and BiTE-redirected T cells against 2.0 × 10^4^ target cells was measured by ELISA. Briefly, anti-human IFN-γ capture mAb (clone 1D1K) was coated onto a Nunc MaxiSorp flat-bottom 96-well plate (Thermo Fisher Scientific) overnight at 4 °C. Collected supernatants were added to the plate and incubated for 2 hours at room temperature. The plate was then incubated with biotin-conjugated anti-human IFN-γ detection mAb (clone 7-B6-1) for 1 hour at room temperature. Then, streptavidin-conjugated alkaline phosphatase was added to each well. One-step pNPP solution (Thermo Fisher Scientific) was utilized for detection. The absorbance at 405 nm was measured by FlexStation3 (Molecular Devices).

### Quantitative analyses of *NY-ESO*-*1* mRNA

Total RNA and cDNA were prepared as described previously^58^. *NY-ESO-1* mRNA was measured by quantitative real-time PCR (qRT-PCR) using QuantiTect SYBR Green PCR kit (Qiagen). *Human hypoxanthine phosphoribosyltransferase 1 (hHPRT1)* mRNA was utilized as an internal control. *NY-ESO-1* mRNA expression was normalized to the levels of *hHPRT1* mRNA, and the relative expression values were calculated using the 2^−ΔΔCt^ method. The following primer sets were used for the experiments: *NY-ESO-1* forward, TTCTGAAGGAGTTCACTGTGTCC, and reverse, AGGGAGGCTGAGCCAAAAAC; *hHPRT1* forward, GGCAGTATAATCCAAAGATGGTCAA, and reverse, GTCAAGGGCATATCCTACAACAAAC.

### Immunoblotting assays

Immunoblotting assays were performed as described previously^59,60^. Cells were extracted with lysis buffer (20 mM Tris-HCL, pH 7.5, 1 mM EDTA, 150 mM NaCl, 1% Triton X-100, 0.1% SDS, 1 mM phenylmethyl sulfonyl fluoride, and 1 μg/mL aprotinin). After centrifugation at 12,000 rpm for 20 minutes at 4 °C, the supernatant was collected as the lysate. Purified BiTEs were used directly for immunoblotting. Following SDS-PAGE, protein was transferred to a polyvinylidene difluoride membrane and incubated with a blocking buffer (Nakarai Tesque). The membranes were incubated with the indicated primary antibodies (anti-NY-ESO-1 mAb: clone E978, anti-β-actin mAb: clone AC-15, anti-His mAb: clone AD1.1.10) overnight at 4 °C, washed and incubated with horseradish peroxidase-conjugated goat anti-mouse IgG (Fc specific) secondary antibody (Sigma Aldrich) for 1 h at room temperature. After washing the membrane, the signal was detected using enhanced chemiluminescence (GE Healthcare).

### Cytotoxicity assays

Standard cytotoxicity assays were performed as described previously^56^. Briefly, 5.0 × 10^3^ target cells were labeled with chromium-51 (^51^Cr) for 1.5 hours at 37 °C. CAR- and BiTE-redirected T cells were incubated with target cells at various E/T ratios for 5 hours (CAR) and 16 hours (BiTE), respectively, unless otherwise specified. The supernatants were collected and their cpm counts were measured. The percentage of specific lysis was calculated as follows: (experimental release cpm − spontaneous release cpm)/(maximal release cpm − spontaneous release cpm) × 100 (%).

### *In vivo* murine experiments

All the murine experiments in this study were approved by the Ehime University Animal Care Committee. All methods were performed in accordance with the relevant guidelines and regulations. Five-week-old NOD/Shi-scid,IL-2RγΚΟ Jic (NOG) mice were purchased from *In-Vivo* Science Inc. and subjected to 1.5 Gy of irradiation. Then, two million U266/SLR cells were intravenously injected into NOG mice. After engraftment, five million redirected T cells, control T cells, or T cells with BiTEs were intravenously injected into each mouse. Tumor sizes were measured by AEQUORIA-2D/8600 bioluminescence imaging assays (Hamamatsu Photonics).

### *In silico* analyses

The ScanProsite tool (http://prosite.expasy.org/scanprosite/) was utilized to search for human-derived peptide sequences homologous to NY-ESO-1_157_ peptide (SLLMWITQC) based on the results of alanine scanning assays. GeneCards (https://www.genecards.org/) was used to search for the expression of proteins containing peptides homologous to NY-ESO-1_157_. The binding affinity of peptides for HLA-A*02:01 was predicted by netMHC4.0 (http://www.cbs.dtu.dk/services/NetMHC/).

### Statistical analyses

Statistical analyses were performed with GraphPad Prism 6.0 h. Welch’s t test (two-sided) or paired t test (two-sided) was employed to examine whether two groups were significantly different for a given variable. Comparisons between more than two groups were performed by two-way ANOVA with Sidak’s multiple comparisons test. P values of <0.05 were considered statistically significant.

## Supplementary information

Supplementary Information

## Data Availability

All data supporting the conclusions of this study are included in the manuscript and its supplementary files, or are available from authors on request.
