# Supplementary material for: Direct comparison of target-reactivity and cross-reactivity induced by CAR- and BiTE-redirected T cells for the development of antibody-based T-cell therapy
Source: Sci Rep. 2019 Sep 16;9:13293. doi: 10.1038/s41598-019-49834-2 (PMC6746725; doi:10.1038/s41598-019-49834-2)
Supplement: Supplementary file 1 — Supplementary Information [file 41598_2019_49834_MOESM1_ESM.pdf]

Direct comparison of target-reactivity and cross-reactivity induced by CAR- and BiTE-redirected T cells for the development of antibody-based T-cell therapy

Masaki Maruta[1], *Toshiki Ochi[1,2], Kazushi Tanimoto[1], Hiroaki Asai[1], Takashi Saitou[3], Hiroshi Fujiwara[1], Takeshi Imamura[3], Katsuto Takenaka[1], Masaki Yasukawa[2]

[1]Department of Hematology, Clinical Immunology, and Infectious Diseases, Ehime University Graduate School of Medicine, Toon, Ehime, Japan 791-0295; [2]Division of Immune Regulation, Proteo-Science Center, Ehime University, Toon, Ehime, Japan 791-0295; [3]Department of Molecular Medicine for Pathogenesis, Ehime University Graduate School of Medicine, Toon, Ehime, Japan 791-0295.

*Correspondence should be addressed to T.O.

Contact Information

Toshiki Ochi, MD, PhD

Department of Hematology, Clinical Immunology and Infectious Diseases,

Ehime University Graduate School of Medicine

Shitsukawa, Toon, Ehime 791-0295, Japan

Phone: +81-89-960-5296

Fax: +81-89-960-5299

E-mail: ochi.toshiki.eg@ehime-u.ac.jp

Supplementary Figure S1

a

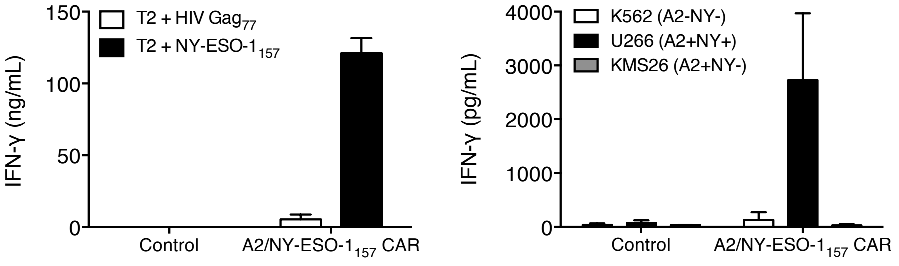

b

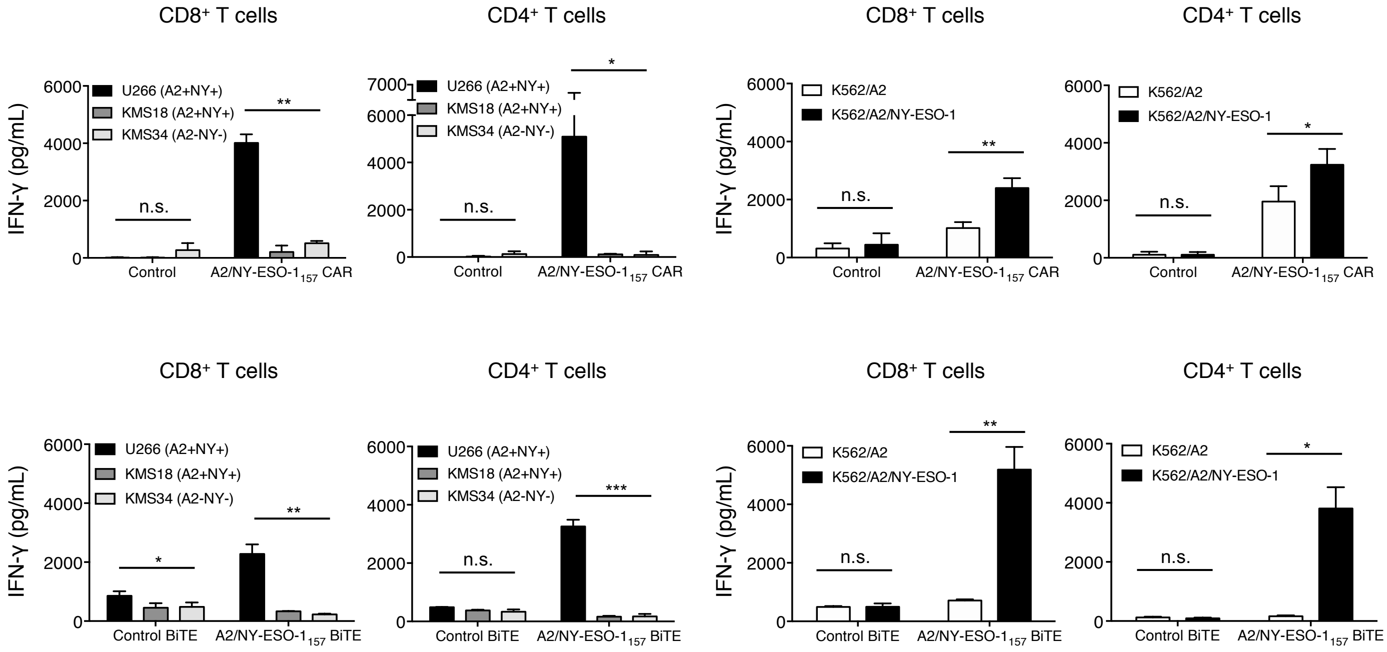

Supplementary Figure S1: A2/NY-ESO-1$_{157}$-specific CAR- and BiTE-redirected T cells recognize HLA-A2^{+}NY-ESO-1^{+} myeloma cells.

(**a**) A2/NY-ESO-1$_{157}$ CAR-redirected T cells established from donor 2 were incubated with the indicated target cells. The culture supernatants were collected and IFN-γ concentration was measured by ELISA assays. The experiments were conducted in triplicate, and error bars demonstrate the SD. (**b**) A2/NY-ESO-1$_{157}$ CAR-redirected CD8^{+} T cells and CD4^{+} T cells were incubated with the indicated target cells (top). Peripheral blood CD8^{+} T cells and CD4^{+} T cells were also incubated with the same target cells in the presence of 5 µg/mL NY-ESO-1$_{157}$ BiTE or control BiTE (bottom). IFN-γ concentration in the culture supernatants was measured similarly to (**a**). The experiments were conducted in triplicate, and representative data obtained from donor 1 are displayed. Error bars depict the SD. *, P<0.05; **, P<0.01; ***, P<0.001; n.s., not significant.

Supplementary Figure S2

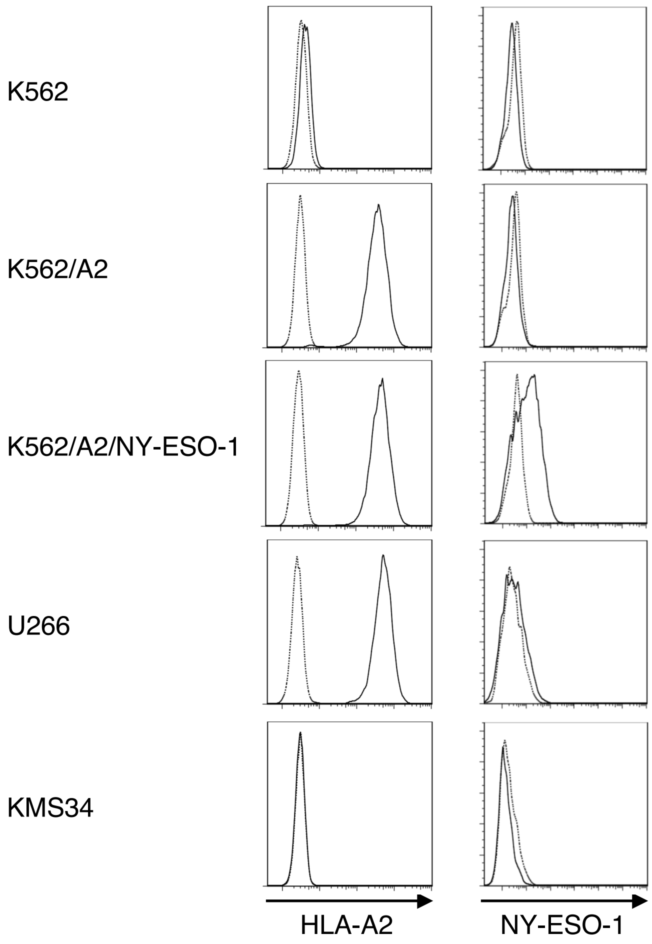

Supplementary Figure S2: Expression of HLA-A2 and NY-ESO-1 in target cells. HLA-A2 and NY-ESO-1 expression in 5 different cell lines used as the target cells in functional assays using CAR- and BiTE-redirected T cells was confirmed. These cell lines were stained with anti-HLA-A2 mAb (left). To stain NY-ESO-1, the cells were fixed and permeabilized, followed by staining with anti-NY-ESO-1 mAb (right). Dotted lines show the isotope control. The experiments were repeated 3 times and representative staining data are shown.

Supplementary Figure S3

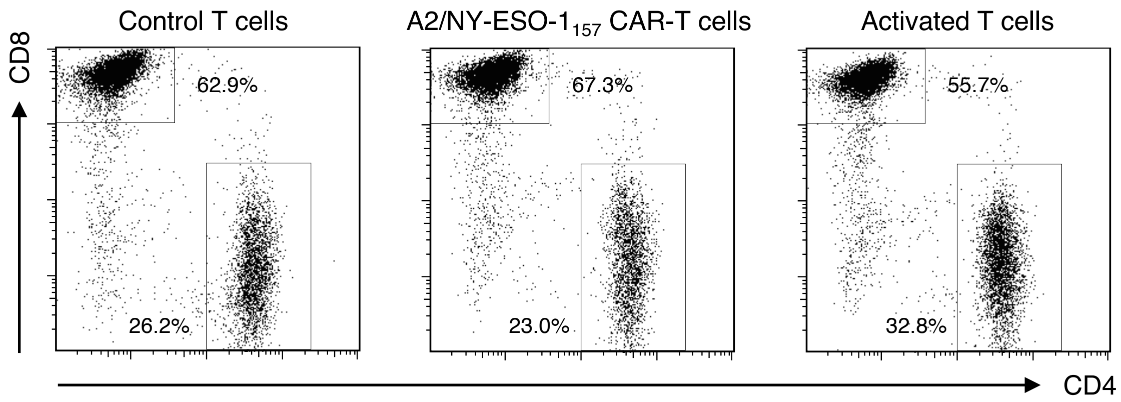

Supplementary Figure S3: CD4/CD8 ratios of CAR-T cells and similarly activated T cells used for *in vivo* experiments.

Human peripheral blood T cells were stimulated as described in Materials and Methods. CD4/8 ratios obtained from control T cells (left), CAR-T cells (middle), and activated T cells (right) that were used for the *in vivo* side-by-side experiments shown in Fig. 6 are depicted.

Supplementary Figure S4

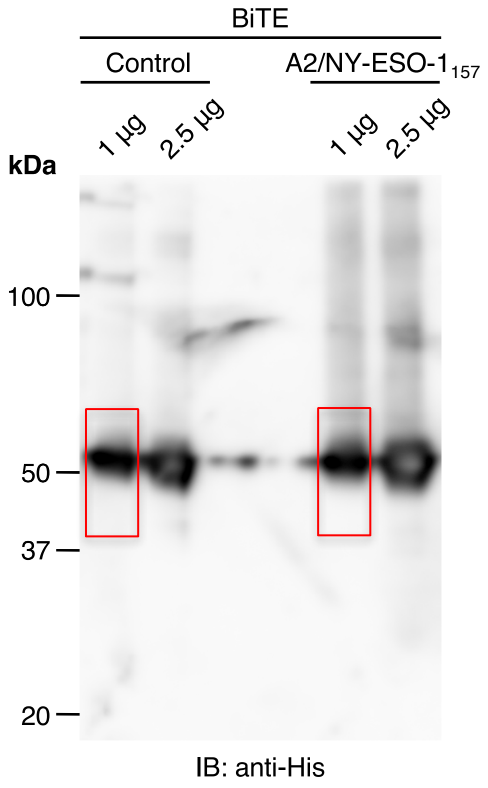

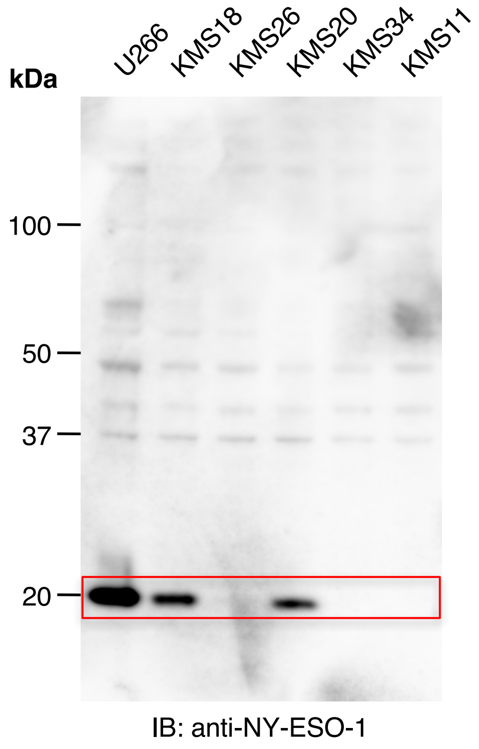

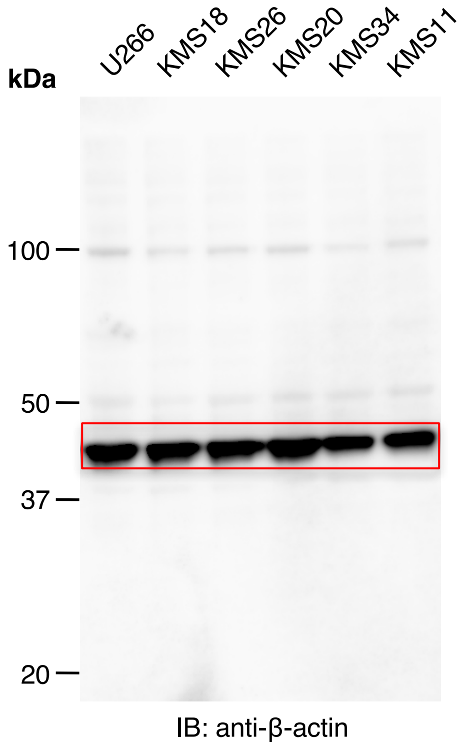

Supplementary Figure S4: The full-length western blotting images.
The areas indicated as red rectangles are displayed in main Figures.
(see Fig. 2a and Fig. 3).

Supplementary Table S1

Protein	Sequence	Binding affinity (nM)
NY-ESO-1 (wild type)	SLLMWITQC	666.69
NY-ESO-1 (heteroclitic)	SLLMWITQV	6.13
Probable E3 ubiquitin-protein ligase HERC1 (HERC1)	ILGMWIPEV	4.71
Solute carrier family 13 member 2 (SLC13A2)	FLSMWISNT	28.28
Taste receptor type 2 member 8 (TA2R8)	YLNMWITTC	222.60
Importin-13 (IPO13)	LLSMWIKEA	215.18
Solute carrier family 13 member 5 (SLC13A5)	LLSMWISNT	348.31
Lysophospholipase-like protein 1 (LYPLAL1)	GLRMWIKQV	1859.63
Ewing’s tumor-associated antigen 1 (ETAA1)	MLDMWIGET	147.04
Thioredoxin-related transmembrane protein 1 (TMX1)	QLSMWIRTC	6051.17
Mitochondrial inner membrane protein (COX18)	QLPMWIFMS	1646.38

Supplementary Table S1: A list of peptides homologous to NY-ESO-1$_{157}$.
Based on the peptide sequence motif (xLxMWIxxx) revealed by alanine scanning assays, 9 different peptides homologous to NY-ESO-1$_{157}$ were synthesized. Binding affinity to HLA-A*02:01 was predicted by netMHC4.0 (http://www.cbs.dtu.dk/services/NetMHC/).
